# Genomic and Phenotypic Analysis of Multidrug-Resistant *Acinetobacter baumannii* Clinical Isolates Carrying Different Types of CRISPR/Cas Systems

**DOI:** 10.3390/pathogens10020205

**Published:** 2021-02-13

**Authors:** Marina Tyumentseva, Yulia Mikhaylova, Anna Prelovskaya, Aleksandr Tyumentsev, Lyudmila Petrova, Valeria Fomina, Mikhail Zamyatin, Andrey Shelenkov, Vasiliy Akimkin

**Affiliations:** 1 Central Research Institute of Epidemiology, Novogireevskaya str., 3a, 111123 Moscow, Russia; tyumentseva@cmd.su (M.T.); mihailova@cmd.su (Y.M.); prelovskaya@cmd.su (A.P.); tymencev@cmd.su (A.T.); vgakimkin@yandex.ru (V.A.); 2 National Medical and Surgical Center named after N.I. Pirogov, Nizhnyaya Pervomayskaya str., 70, 105203 Moscow, Russia; lutix85@yandex.ru (L.P.); med_2006@mail.ru (V.F.); mnz1@yandex.ru (M.Z.)

**Keywords:** *Acinetobacter baumannii*, whole genome sequencing, antibiotic resistance, MDR, CRISPR/Cas systems

## Abstract

*Acinetobacter baumannii* is an opportunistic pathogen being one of the most important causative agents of a wide range of nosocomial infections associated with multidrug resistance and high mortality rate. This study presents a multiparametric and correlation analyses of clinical multidrug-resistant *A. baumannii* isolates using short- and long-read whole-genome sequencing, which allowed us to reveal specific characteristics of the isolates with different CRISPR/Cas systems. We also compared antibiotic resistance and virulence gene acquisition for the groups of the isolates having functional CRISPR/Cas systems, just CRISPR arrays without cas genes, and without detectable CRISPR spacers. The data include three schemes of molecular typing, phenotypic and genotypic antibiotic resistance determination, as well as phylogenetic analysis of full-length cas gene sequences, predicted prophage sequences and CRISPR array type determination. For the first time the differences between the isolates carrying Type I-F1 and Type I-F2 CRISPR/Cas systems were investigated. *A. baumannii* isolates with Type I-F1 system were shown to have smaller number of reliably detected CRISPR arrays, and thus they could more easily adapt to environmental conditions through acquisition of antibiotic resistance genes, while Type I-F2 *A. baumannii* might have stronger “immunity” and use CRISPR/Cas system to block the dissemination of these genes. In addition, virulence factors *abaI, abaR*, *bap* and *bauA* were overrepresented in *A. baumannii* isolates lacking CRISPR/Cas system. This indicates the role of CRISPR/Cas in fighting against phage infections and preventing horizontal gene transfer. We believe that the data presented will contribute to further investigations in the field of antimicrobial resistance and CRISPR/Cas studies.

## 1. Introduction

*Acinetobacter baumannii* is an important opportunistic pathogen responsible for a wide range of hospital-acquired infections (HAI), and is associated with respiratory infections, bacteremia, meningitis, and wound infections [1]. The percentage of carbapenem- and multidrug-resistant strains causing nosocomial outbreaks in various regions of the world is growing exponentially [2]. Recently, the World Health Organization has included carbapenem-resistant *A. baumannii* in the Global List of critical priority level for scientific research and the development of new antibiotics [3].

The main features of *A. baumannii* are the intrinsic multidrug resistance; the ability to form biofilms (both on the tissues of a living organism and on polymeric materials used in medicine); the presence of a signaling system “quorum-sensing”, which makes it possible to enhance the protection of bacteria against antibiotics, disinfectants, and the human immune system [4]. *A. baumannii* is characterized by high epidemic potential and quickly forms hospital clones. For the epidemiological surveillance of this important species, two multilocus sequence typing schemes (MLST), usually referenced as Oxford and Pasteur and showing different levels of resolution, have been established [5,6].

Clustered regularly interspaced short palindromic repeat (CRISPR) arrays and CRISPR-associated genes (cas) constitute bacterial adaptive immune systems and function as a variable genetic element [7]. CRISPRs are bacterial loci whose dynamic nature has allowed them to become ideal targets for molecular subtyping. Each CRISPR/Cas locus includes a strain-specific array of spacers that has expanded and diversified over time. Due to their dynamic nature, comparative analysis of the spacer arrays has successfully been used for subtyping isolates from several Gram-positive and Gram-negative bacteria, including *Mycobacterium tuberculosis*, *Yersinia pestis* and the plant pathogen *Erwinia amylovora* [8]. Arrays of spacers were found to be highly polymorphic in *Salmonella*, and a strong correlation was detected between polymorphisms in the arrays and the serotypes [8,9]. Multiple reports have suggested that CRISPR/Cas systems may play a major role in controlling horizontal gene transfer and, consequently, the dynamics of antibiotic resistance gene acquisition in *Pseudomonas aeruginosa* [10,11].

Two CRISPR/Cas systems have recently been found in the genomes of several *A. baumannii* strains [12,13]. Studies have reported that trailer end spacers of CRISPR are generally conserved among different isolates and can be used to anchor clusters and detect common ancestors of the arrays and, probably, of the isolates themselves [14]. More and more data are accumulating indicating that the role of CRISPR/Cas is not limited to adaptive immunity. It has been shown that these systems regulate the expression of many bacterial genes affecting the virulence of pathogenic bacteria and group behavior, and also participate in DNA repair and accelerate the evolution of genomes [15].

*A. baumannii* is one of the most common causative agents of HAI infections in Russia [16]. In the present study, we provide the comprehensive data based on second- and third-generation (long-read) sequencing for 12 clinical isolates of *A. baumannii* obtained from Moscow multidisciplinary medical center. We used Pasteur MLST typing scheme complemented by capsular (KL) and oligosaccharide (OCL) loci. We also explored genes associated with antimicrobial resistance and conducted sequenced-based analysis of CRISPR arrays and CRISPR/Cas systems. The abundance of the latter was shown to be associated with antibiotic susceptibility in *Streptococcus pyogenes*, *Escherichia coli* and *Klebsiella pneumoniae* [17,18,19]. Comparative analysis allowed determining the phylogenetic relationship between the cas1 and other CRISPR/Cas loci from the isolates studied and a collection of publicly available global *A. baumannii* strains. Additionally, we assessed specific features of *A. baumannii* isolates having different types of CRISPR/Cas systems and compared the groups of the isolates with and without functional CRISPR/Cas systems.

## 2. Results

### 2.1. Isolate Typing

MLST is commonly used as a method of choice for epidemiological surveillance of pathogenic bacteria, if such a scheme exists for the species under study. Additional typing of *A. baumannii* may be performed by using capsule synthesis loci (K-loci) [20] or lipooligosaccharide outer core loci (OCL) [21]. The results of the isolate typing are presented in Table 1. The isolates belonged to eight different sequence types according to Pasteur MLST scheme. It was chosen for typing since, according to the Oxford scheme, five isolate possessed undetectable sequence types (STs) with duplicated *gdhB* locus. ST2 was found in four isolates, but they have different KL types that was in line with our goal to cover the representative population of all hospital isolates. It was also interesting that two isolates, CriePir306 and CriePir308, were obtained from the same patient (A9), but had different STs, thus indicating simultaneous infection by multiple strains. The isolate CriePir307 possessed novel ST, which allelic profile was cpn60 (8), fusA (1), gltA (16), pyrG (3), recA (6), rplB (2), rpoB (3). We have submitted this isolate to the Institut Pasteur MLST system (http://bigsdb.pasteur.fr, accessed on 20 December 2020), and new profile ST1487 was assigned to this allele combination.

In addition, the isolates were assigned to nine different KL-types and four OCL-types. The most prevalent KL-types were KL9 and KL2, which were totally revealed in about 36% of the isolates from NCBI WGS database [21]. Interestingly, CriePir298 had a recently described KL116 type [22], which is rather rare [21]. The most frequent OCL was OCL1, which was also the most common type in NCBI WGS database [21]. In general, currently 92 KL and 12 OCL types are available, so that our dataset shows high variability in lipooligosaccharide loci types and relatively low variability in capsule synthesis loci types.

It is worth noting that KL-type provides additional classification narrowing in comparison to sequence type alone. For example, CriePir33 forms standalone clade since it possesses the KL-type (KL3) different from its neighbors’ type (KL2), although they share the same ST (ST78). The same is observed for CriePir87—it has KL33, while its neighbors with identical ST (ST2) are characterized by KL2. Thus, such a hybrid typing scheme that we have recently applied for *K. pneumoniae* [23], proved to be applicable for precise distinguishing of the isolates in clinical settings, which is an important task in epidemiological surveillance and infection prevention.

Additional typing performed using cgMLST loci complied well with MLST and KL analysis. cgMLST profiles for the isolates studied are presented in Table S1, and cgMLST-based tree is shown in the Figure S11.

### 2.2. Antibiotic Susceptibility Testing and Antimicrobial Resistance Gene Identification

**Figure 1:**
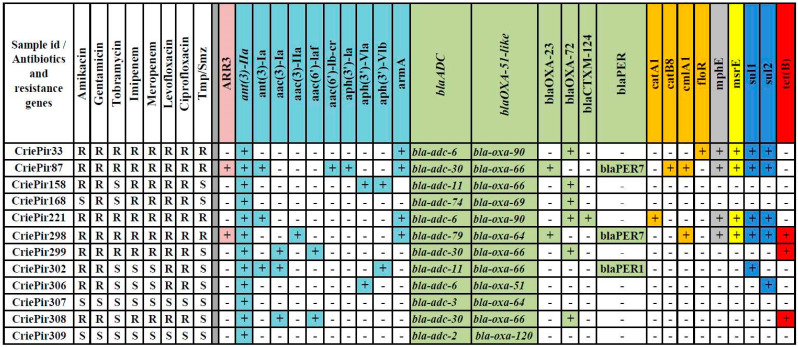
Antibiotic resistance profiles and distribution of antibiotic resistance determinants of clinical *A. baumannii* isolates studied. Tmp/Smz—trimethoprim/sulfamethoxazole; “+” and “−“ indicates the presence and absence of antibiotic resistance determinant, respectively; gene clusters for resistance to different groups of antibiotics are highlighted by different colors. Intrinsic *ant(3″)-IIa, blaADC* and *blaOXA-51-like* genes are presented at the beginning of corresponding groups and are italicized.

The results of phenotypic analysis are presented in Figure 1. Four isolates (CriePir33, CriePir87, CriePir221, and CriePir298) were resistant to all antibiotics tested. The isolate CriePir299 was susceptible to trimethoprim/sulfamethoxazole only. CriePir168 was susceptible to three antibiotics—amikacin, tobramycin, and trimethoprim/sulfamethoxazole, while other two isolates (CriePir302 and CriePir306) were susceptible to meropenem, imipenem and trimethoprim/sulfamethoxazole in addition to tobramycin. The isolates CriePir307 and CriePir309 were susceptible to all antibiotics tested.

We examined the distribution of acquired antibiotic resistance genes among the clinical isolates studied. The results of resistance determinant annotation are presented in Figure 1. The spectrum of identified resistance determinants included gene clusters providing resistance to aminoglycosides, beta-lactams, chloramphenicol and sulfonamides. Some isolates were characterized by the presence of genes determining resistance to rifampicin, erythromycin and tetracycline.

Different alleles of intrinsic cephalosporinase-encoding gene *bla_ADC_* were revealed in all isolates studied. The isolates possessed a wide variety of oxacillinase genes (*bla_OXA_*, eight genes), most of which encoded carbapenem-hydrolyzing class D β-lactamases (CHDLs) for those *A. baumannii* had been previously found to be an original host [24]. CriePir221 also encoded extended spectrum β-lactamase (ESBL) (*bla_CTX-M-124_* gene), while CriePir302 encoded PER-1, and CriePir87 and CriePir298 encoded PER-7 ESBLs, respectively, which were known to possess an increased activity toward broad-spectrum cephalosporins in *A. baumannii* [25]. The isolate CriePir87 was the one having the maximal number of acquired antibiotic resistance genes (13 genes of 23 identified).

Comparative analysis shows that *bla_OXA-72_* and *bla_OXA-23_* represent carbapenemase genes that are likely to confer meropenem and imipenem resistance for 8 out of 12 isolates studied. For the isolates sequenced on MinION, the location of *bla_OXA-72_* was found to be on plasmid (CriePir168), while the bla_OXA-23_ location was on chromosome (CriePir298). These findings comply with the results described previously [26,27].

### 2.3. CRISPR Arrays and CRISPR/Cas Systems

Ten isolates from our study included confirmed CRISPR repeats sequences determined by CRISPRCasFinder [28]. Three isolates (CriePir168, CriePir298 and CriePir307) carried CRISPR loci with encoded Cas proteins which represent an important component for the functioning of a putative CRISPR/Cas system. It is worth noting that one isolate (CriePir307) having such putative system was susceptible to all antibiotics tested, while the other two isolates were MDR. These three isolates also possessed different integrated plasmid sequences. All other isolates except two (CriePir158 and CriePir299, which did not have any CRISPR spacers) possessed CRISPR arrays but lacked any detectable cas genes. Among them, the isolate CriePir309 draws attention due to the presence of many CRISPR spacers with plasmid localization and the lack of Cas proteins. The highest homology of its spacers was revealed to the following Acinetobacter phage sequences: phiAC-1, vB_AbaM_IME284, AB1, YMC1112R2315 and WCHABP1. The number and characteristics of CRISPR spacers revealed in the isolates studied are presented in Table S2.

**Figure 2:**
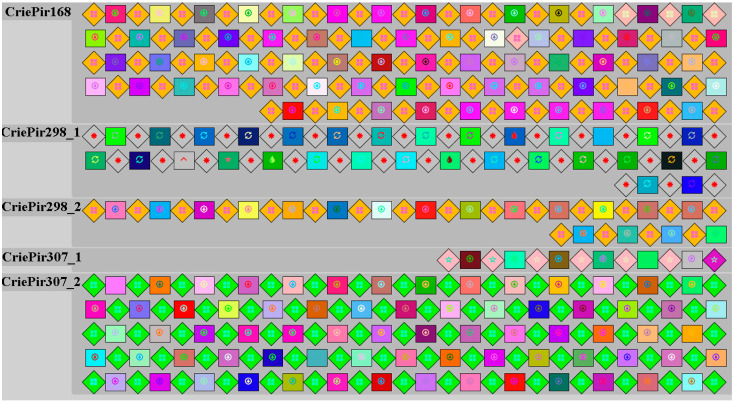
Spacer (squares) and repeat (diamonds) composition for clinical *A. baumannii* isolates having putative Type I-F CRISPR/Cas systems.

Putative CRISPR/Cas systems found in CriePir168, CriePir298 and CriePir307 belonged to Type I-F. Distinct assortment of spacers harbored by each of the three isolate is presented in Figure 2.

The numbers of spacers in the CRISPR locus of the isolates CriePir168, CriePir298 and CriePir307 are respectively 73, 47 and 85. Spacer numbers vary without any relationship to plasmid size or the presence/absence of antibiotic resistance genes. Spacer composition varied between isolates. The genome of CriePir168 isolate contains spacers homologous to prophage sequences; the highest homology was found with Acinetobacter phage vB_AbaS_TRS. Prophage sequences comprising CriePir298 CRISPR array included Acinetobacter phages AM106, Ab105-3phi and Pseudomonas phage AUS531phi. The CRISPR spacers of CriePir307 isolate matched with Acinetobacter phages Ab105-2phi, vB_AbaS_TRS1 and YMC1111R3177. The CRISPR arrays analyzed also included plasmid sequences, namely, CriePir168 possessed CRISPR spacers matching Acinetobacter plasmids pDA33382-2-2, pAba7804b and pAB3, the isolate CriePir298 carried spacers homologous to plasmid p2014S01-097-2 and unnamed *A. baumannii* plasmid of the strain B11911, while CriePir307 was characterized by CRISPR spacers matching unnamed *A. baumannii* plasmid of the strain CIAT758, pAba9201a, p2014S01-097-2 and pTG31302.

Direct repeats of CriePir168 and CriePir298 belong to structure motif 6 and represent sequence family 8, while direct repeats of CriePir307 belong to structure motif 4 and represent unclassified sequence family (Figure S1).

The CRISPR/Cas loci of the isolates CriePir168 and CriePir298 were similar and consisted of six genes encoding Cas6 endoribonuclease, three Csy proteins (Csy3, Csy2 and Csy1), Cas3/Cas2 helicase/RNase and Cas1 endonuclease in the vicinity of CRISPR arrays. The CriePir307 isolate carried Cas1, Cas3/Cas2, Cas6, and two Csy proteins (Csy2 and Csy3) downstream of the CRISPR-array. An internal deletion was detected in the locus of this isolate, resulting in the absence of Csy1. None of the genes of described CRISPR/Cas loci contained premature stop codons, and the genes of anti-CRISPR proteins were not found.

**Figure 3:**
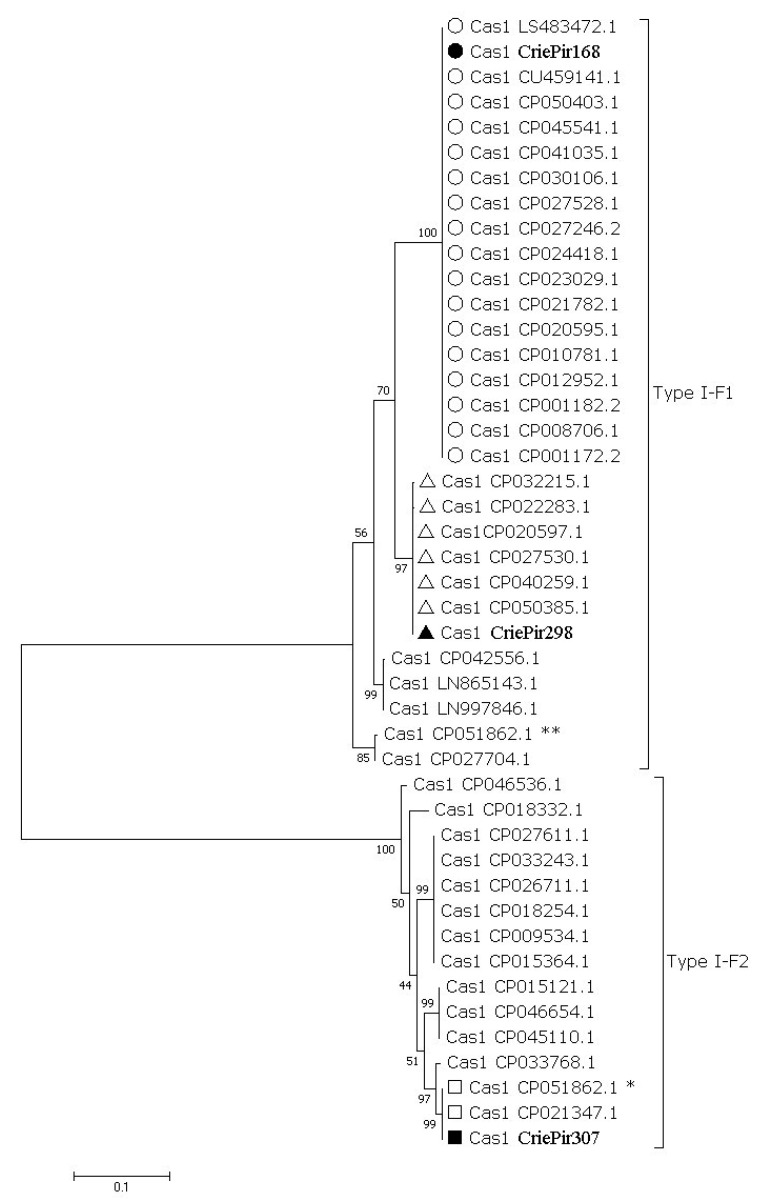
Maximum-likelihood phylogenetic tree of full-length cas1 gene sequences of *A. baumannii* detected in clinical departments of multidisciplinary medical center in Moscow, Russia during the period of 2017-2019. Bootstrap test (1000 replicates) was used, and bootstrap values are shown at the branch nodes. The sequences identified in this study are marked with black circle, square and triangle, respectively. Reference sequences belonging to the clades containing the sequences identified in this study are marked with transparent circles, squares and triangles, respectively. Sequences identified in this study are indicated by the isolate names, reference sequences—by GenBank accession number. Asterisks (*, **) indicate two different cassettes of cas genes found in one of the reference isolates.

Reference isolates, as well as our isolates, were characterized by genotypic antibiotic resistance profiles, plasmid number, the presence of prophage sequences, and CRISPR array type. Maximum likelihood phylogenetic tree of full-length cas1 gene sequences showed that *A. baumannii* isolates CriePir168 and CriePir298 belonged to Type I-F1 CRISPR/Cas system (30 out of 45 sequences), while CriePir307 belonged to Type I-F2 (15 out of 45 sequences) (Figure 3) [29,30]. Topology of the isolates studied on the phylogenetic trees constructed for full-length cas gene sequences was identical, e.g., the same sequences represented clades containing Type I-F1 and Type I-F2 isolates (Figures S2–S6). For simplicity, the names of our isolates are shortened just to ‘P’ instead of ‘CriePir’ on these figures.

To assess the characteristics of *A. baumannii* CRISPR/Cas system, a list of 242 reference isolates (Table S3) was composed according to CRISPRCasdb. All isolates were divided into four groups according to the CRISPR/Cas system present—“No CRISPR/No Cas”, “CRISPR/No Cas”, “CRISPR/Cas Type I-F1” and “CRISPR/Cas Type I-F2” (Table S3). To reveal the correlation between *A. baumannii* MDR and CRISPR/Cas system reference isolates, as well as our isolates, were characterized by genotypic antibiotic resistance profiles, plasmid number and the presence of genes encoding virulence factors.

**Figure 4:**
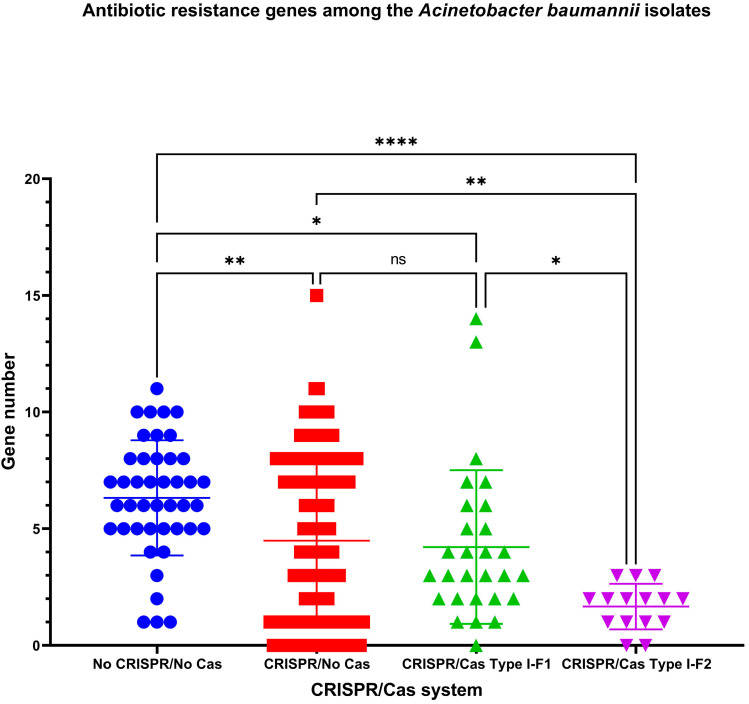
The number of antibiotic resistance genes among *A. baumannii* isolates. Ns – not significant; *, ** and **** indicate the increasing levels of significance.

The number of antibiotic resistance genes differs between the analyzed groups of the isolates (Figure 4). The highest number of antibiotic resistance genes (Mean ± SD, 6.326 ± 2.466) was found in “No CRISPR/No Cas” *A. baumannii* isolates, the lowest—in “CRISPR/Cas Type I-F2” isolates (Mean ± SD, 1.667 ± 0.976). The number of antibiotic resistance genes in “CRISPR/No Cas” and “CRISPR/Cas Type I-F1” did not differ significantly (Figure 4).

Interestingly, the lowest number of plasmids was found in “No CRISPR/No Cas” *A. baumannii* isolates (Mean ± SD, 1.349 ± 1.378). It differs significantly only from “CRISPR/Cas Type I-F1” group having the highest number of plasmids among the analyzed groups (Mean ± SD, 2.393 ± 2.132) (Figure S7).

All *A. baumannii* isolates had similar sets of genes encoding virulence factors. Common set consists of 40 genes: abaI, abaR, adeF, adeG, adeH, bap, barA, barB, basA, basB, basC, basD, basF, basG, basI, basJ, bauA, bauB, bauC, bauD, bauE, bauF, bfmR, bfmS, csuA, csuA/B, csuB, csuC, csuD, csuE, entE, ompA, pgaA, pgaB, pgaC, pgaD, plc, and plcD. Significant differences were found in frequencies of occurrence of virulence factors abaI, abaR, bap, basH, bauA, and bfmR (Table S4). AbaI (N-acyl-L-homoserine lactone synthetase) occurs more frequently in “No CRISPR/No Cas” *A. baumannii* isolates and the analyzed groups can be arranged in descending order as “No CRISPR/No Cas” > “CRISPR/No Cas”, “CRISPR/Cas Type I-F1” > “CRISPR/Cas Type I-F2”. AbaR (DNA-binding HTH domain-containing protein) was found less frequently in “CRISPR/Cas Type I-F2” *A. baumannii* isolates. It should be noted that “CRISPR/Cas Type I-F1” *A. baumannii* isolates analyzed in our study completely lack bap gene that encodes biofilm-associated protein. BasH (non-ribosomal peptide biosynthesis thioesterase BasH) was revealed slightly less often in “CRISPR/No Cas” isolates. Moreover, bauA encoding TonB-dependent siderophore receptor BauA was underrepresented in “CRISPR/Cas Type I-F1” group of *A. baumannii* isolates (in one out of 28 isolates). BfmR (biofilm-controlling response regulator) is the only gene slightly underrepresented in “No CRISPR/No Cas” group (Table S4).

To assess the characteristics of *A. baumannii* putative CRISPR/Cas system containing both CRISPR arrays and cas genes, reference isolates, as well as our isolates, were further characterized. Additionally, the presence of prophage sequences, and CRISPR array types were assessed.

**Figure 5:**
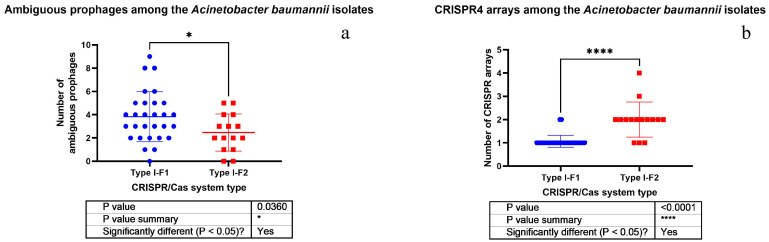
Comparison of *A. baumannii* isolates having different CRISPR/Cas systems by the number of ambiguous prophages (**a**) and CRISPR4 arrays (**b**).

It was found that the isolates possessing Type I-F1 CRISPR/Cas system had significantly more antibiotic resistance genes (Figure 4) and ambiguous prophage sequences in their genomes (Figure 5a). Moreover, Type I-F1 CRISPR/Cas system bearing isolates had significantly less CRISPR4 arrays (the most reliably predicted CRISPRs) (Figure 5b) [31].

**Figure 6:**
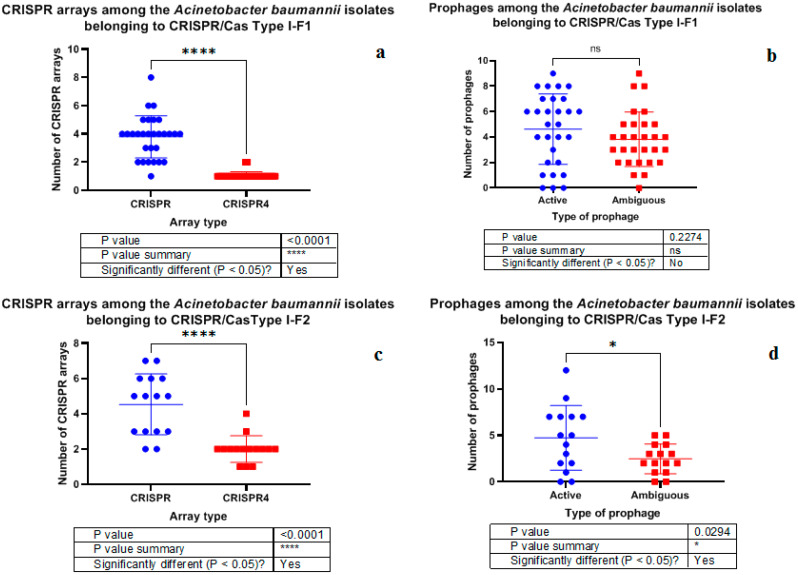
Comparison of *A. baumannii* isolates carrying Type I-F1 and Type I-F2 systems by CRISPR arrays (**a**,**c**) and prophage sequences (**b**,**d**).

It should be noted that no significant differences in plasmid number, active prophage sequence number and low evidence CRISPR array number were detected in the isolates bearing either Type I-F1 or Type I-F2 CRISPR/Cas system (Figures S8–S10). Furthermore, Type I-F1 CRISPR/Cas system of *A. baumannii* had significantly higher number of low evidence CRISPR arrays than CRISPR4 arrays (Figure 6a), and no significant difference was found in active versus ambiguous prophage sequence numbers among them (Figure 6b). At the same time, Type I-F2 CRISPR/Cas system of *A. baumannii* had significantly more low evidence CRISPR arrays and active prophage sequences (Figure 6c and Figure 6d, respectively).

**Figure 7:**
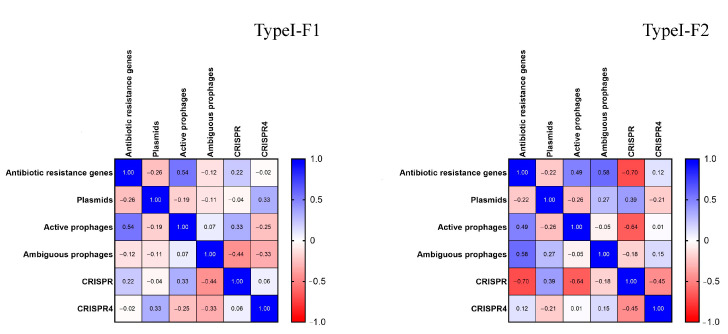
Correlation matrices for Type I-F1 and Type I-F2 CRISPR/Cas system data sets.

Additionally, we conducted correlation analysis. Normalization of the data sets “Antibiotic resistance genes”, “Plasmids”, “Active prophages”, “Ambiguous prophages”, “CRISPR” and “CRISPR4” for both Type I-F1 and Type I-F2 was performed and nonparametric Spearman correlation was calculated, and correlation matrices for each pair of data sets were constructed. It was shown that Type I-F1 CRISPR/Cas systems had moderate positive correlation (rs = 0.54, *p* = 0.003) between the number of active prophage sequences and number of antibiotic resistance genes and low negative correlation (rs = −0.44, *p* = 0.02) between the number of ambiguous prophage sequences and the number of low evidence CRISPR arrays (Figure 7).

Type I-F2 CRISPR/Cas systems had different correlation patterns than those of Type I-F1. Type I-F2 CRISPR/Cas system data sets were characterized by moderate positive correlation (rs = 0.58, *p* = 0.025) between the number of ambiguous prophage sequences and the number of antibiotic resistance genes, and by moderate negative correlation between (i) the number of low evidence CRISPR arrays and the number of antibiotic resistance genes (rs = −0.70, *p* = 0.005) and (ii) the number of low evidence CRISPR arrays and the number of active prophage sequences (rs = −0.64, *p* = 0.011) (Figure 6).

## 3. Discussion

In this study we analyzed 12 clinical isolates of *A. baumannii* which belonged to eight different MLST-based sequence types including the novel ST1487 represented by the isolate CriePir307.We also proposed a hybrid typing scheme (MLST/KL typing) that we recently applied to *K. pneumoniae* [23] for better distinguishing of the isolates *A. baumannii* in clinical settings. Although many typing schemes were already provided for pathogenic bacteria, including the ones based on the nucleotide frequency matrices for genomic sequences [32], CRISPR sequences [33], and regular sequences classification [34], the hybrid typing scheme including MLST, KL, and, possibly, OCL loci seems to be the most promising for the species where all of them are available. Additional typing by cgMLST alleles confirmed the classification provided by hybrid typing scheme (Figure S11).

Ten of 12 isolates expressed an MDR phenotype; four of them were resistant to the whole panel of eight antibiotics belonging to various classes. Long-read sequencing on MinION (Oxford Nanopore Technologies, Oxford, UK) allowed to greatly improve the assembly of four *A. baumannii* genomes (down to one contig for each genome), including the distinguishing of the plasmid sequences. This allowed us to obtain the precise locations of carbapenemase genes for these isolates—*bla_OXA-72_* was found on plasmid for CriePir168, while the bla_OXA-23_ location was revealed on the chromosome for CriePir298, which complies with previous findings [26,27]. In general, the isolates exhibited good compliance between phenotypic and genomic resistance profiles, and no discrepancies were observed for the antibiotics tested. However, the panel was not diverse enough to make global conclusions regarding phenotype-genotype correlations. The data obtained will facilitate future studies of antimicrobial resistance mechanisms in Acinetobacter species.

Additionally, we analyzed CRISPR arrays those were observed in 10 isolates, and putative CRISPR/Cas systems possessed by 3 isolates of our samples and 242 reference isolates according to the CRISPRCasdb. CRISPRs are described in a wide range of prokaryotes [31]. Only 36% of bacteria carry both CRISPR arrays and cas genes. According to CRISPRCasdb, about 20% of representatives of *Acinetobacter* genus and 18% isolates of *A. baumannii* species carry both CRISPR arrays and cas genes.

CRISPR/Cas system of Type I is the most widespread in nature [29] and is characterized by the presence of multi-subunit effector complex. Specific compositions of this complex include 9 subtypes, namely, A, B, C, G, D, E, F1 (previously F), F2 (previously F variant), and F3 [29,30]. The most well-known CRISPR/Cas Type I systems are Type I-E systems, while Type I-F1 CRISPR/Cas system is their closest relative [35]. The Type I-F1 CRISPR/Cas system is characterized by a unique fusion of Cas2 to Cas3 (Cas2/3), which together with Cas1 mediate spacer integration into CRISPR locus [36]. Cas2/3 also degrades foreign DNA, which is targeted by the Type I-F1 cascade (i.e., CRISPR-associated complex for antiviral defense) or Csy (i.e., crRNA-guided surveillance) complex. Csy complex typically consists of four different Cas proteins: Csy1 also known as Cas8f, Csy2 (Cas5f), Cas7f (Csy3), and Csy4 (Cas6f) [37]. It should be noted that Csy1 is missing in Type I-F2 CRISPR/Cas system, but the existing Csy2 containing an additional domain may compensate for the roles of Csy1 [38,39].

*A. baumannii* is known to have Type I-F CRISPR/Cas systems [13], but some species carry type IV variant with genes *csf3*, *csf4* (also known as *dinG*) and *cas6e*, together with CRISPR arrays at both ends [40]. Our study involved *A. baumannii* isolates detected in clinical departments of multidisciplinary medical center in Moscow, Russia, during the period of 2017-2019, as well as reference isolates having active CRISPR/Cas system according to CRISPRCasdb. Approximately one third of *Acinetobacter baumannii* species carry both CRISPR arrays and cas genes belonging to Type I-F2. In attempt to reveal specific features of *A. baumannii* CRISPR/Cas systems, we performed multiparametric analysis of *A. baumannii* genomes including phylogenetic analysis of full-length cas gene sequences, prophage sequence prediction, genomic antimicrobial resistance profile prediction and detection of CRISPR array types.

We found the identical topology of the isolates studied on the phylogenetic trees constructed for full-length cas gene sequences, namely, the same sequences represented the clades containing Type I-F1 and Type I-F2 isolates, which implied a vertical spread of the CRISPR/Cas locus in *A. baumannii*. This fact was also suggested earlier by Karah et al. [13].

In the present study, we have demonstrated clear association of MDR genotype/phenotype of *A. baumannii* with the type of its CRISPR/Cas system. Isolates lacking both CRISPR arrays and active cas genes were shown to have much more antibiotic resistance genes than those having only CRISPR arrays or both CRISPR arrays and cas genes. This complies with the fact that CRISPR/Cas system represents a so-called prokaryotic “immune system” that can fight not only against phage infections, but also against dissemination of antibiotic resistance genes in *A. baumannii*.

Additionally, correlation of *A. baumannii* virulence factors and CRISPR/Cas systems was found. Virulence factors can be horizontally transferred easily among isolates by phage transduction. Several factors, namely *abaI, abaR*, *bap* and *bauA*, are overrepresented in *A. baumannii* isolates lacking CRISPR/Cas system. This indicates the role of CRISPR/Cas in fighting against phage infections and preventing horizontal gene transfer.

For the first time the differences between the isolates carrying Type I-F1 and Type I-F2 CRISPR/Cas systems were investigated. One of the most significant differences between Type I-F1 and Type I-F2 isolates of *A. baumannii* revealed in the present study is the lower number of CRISPR4 arrays in Type I-F1 *A. baumannii* isolates. Lower number of highly confident CRISPR arrays located together with cas genes suggests that “immune system” of Type I-F2 *A. baumannii* isolates is stronger. As a result, we noticed another significant difference between Type I-F1 and Type I-F2 isolates of *A. baumannii*, namely, higher number of antibiotic resistance genes in Type I-F1 *A. baumannii* isolates. This finding may imply that Type I-F1 *A. baumannii* isolates more easily adapt to environmental conditions through acquisition of antibiotic resistance genes, while Type I-F2 *A. baumannii,* presumably, may use CRISPR/Cas system to block dissemination of antibiotic resistance genes. It is worth noting that wild type antimicrobial-susceptible *E. coli* strains carry Type I-F1 CRISPR/Cas system, and it is supposed to interfere with the acquisition of antimicrobial resistance plasmids, maintaining susceptibility in these *E. coli* isolates [18]. The hypothesis that CRISPR/Cas bacterial adaptive immune systems prevent clinical isolates from acquiring antibiotic resistance elements has been widely discussed; however, it has been described in details for the Enterococci only [40]. In the research mentioned it was suggested that type II CRISPR/Cas systems reduced the acquisition of antibiotic resistance and were negatively associated with MDR isolates. In silico analysis of the pan-genome of 2500 *A. baumannii* strains by Mangas et al. [41] showed that they could be divided into two groups having different number of common genes within each group, which could be maintained by CRISPR/Cas systems. The group with these defense systems seems to have specific biofilm genes, and would prevent the acquisition of plasmids and, probably, foreign genes, including resistance elements [41].

Moreover, we have shown that Type I-F1 *A. baumannii* isolates are characterized by the presence of higher number of ambiguous prophage sequences. This fact may also be linked with weakness of their “immune” CRISPR/Cas system, i.e., Type I-F1 *A. baumannii* isolates include less CRISPR4 arrays, and so they are less capable to fight against phage infections via CRISPR/Cas. Presumably, Type I-F1 *A. baumannii* isolates possess distinctive molecular mechanisms to arrest phage infections and to convert an active prophage into ambiguous one through point mutations, genome rearrangements, modular exchanges, invasion by mobile DNA elements, massive DNA deletion, etc.

Despite the fact that we had a small collection of clinical isolates of *A. baumannii*, three of 12 isolates were characterized by different types of CRISPR/Cas systems. Their further analysis, together with 242 reference strains, allowed us to find statistically significant differences. For the first time we showed that *A. baumannii* isolates with Type I-F1 and Type I-F2 CRISPR/Cas system are characterized by different numbers of predicted CRISPR4 arrays, antibiotic resistance genes and ambiguous prophage sequences.

In conclusion, the data obtained could facilitate further investigations in the field of studying the genome dynamics of *A. baumannii* through fast evolving CRISPR/Cas systems, in particular, their role in controlling antibiotic resistance gene transfer and acquisition.

## 4. Materials and Methods

### 4.1. Determination of Antibiotic Susceptibility

All isolates were identified down to a species level by time-of-flight mass spectrometry (MALDI-TOF MS) using the VITEC MS system (bioMerieux, Marcy-l’Étoile, France). The susceptibility was determined by the disc diffusion method using the Mueller-Hinton medium (bioMerieux, Marcy-l’Étoile, France) and disks with antibiotics (BioRad, Marnes-la-Coquette, France), and by the boundary concentration method on VITEK2Compact30 analyzer (bioMerieux, Marcy-l’Étoile, France). The isolates were tested for susceptibility/resistance to the following drugs: amikacin, gentamicin, tobramycin, imipenem, meropenem, levofloxacin, ciprofloxacin and trimethoprim/sulfomethoxazole. The panel of antimicrobial compounds included for testing in this study reflected those agents used for human therapy in Russian Federation. To interpret the results obtained, we used the EUCAST clinical breakpoints, version 11.0 (https://www.eucast.org/clinical_breakpoints/, accessed on 20 December 2020) where available.

### 4.2. DNA Isolation, Sequencing and Genome Assembly

Twelve samples were obtained from 11 patients (6 males and 5 females) in various sources and clinical departments (Table 1) of multidisciplinary medical center in Moscow, Russia during the period of 2017–2019. Patient age ranged from 27 to 62 with a median equal to 52 years. Ten isolates exhibited MDR phenotypes, and two (CriePir307 and CriePir309) were susceptible to all antibiotics tested.

Genomic DNA was isolated with DNeasy Blood and Tissue kit (Qiagen, Hilden, Germany) and used for paired-end library preparation with Nextera™ DNA Sample Prep Kit (Illumina^®^, San Diego, CA, USA) and whole-genome sequencing (WGS) of all 12 isolates on Illumina^®^ Hiseq platform (Illumina^®^, San Diego, CA, USA).

WGS was also performed using the Oxford Nanopore MinION sequencing system (Oxford Nanopore Technologies, Oxford, UK) for 4 out of 12 isolates, which carried CRISPR arrays and/or CRISPR/Cas loci. DNA was used to prepare the MinION library with the Rapid Barcoding Sequencing kit SQK-RBK004 (Oxford Nanopore Technologies, Oxford, UK). The amount of initial DNA used for barcoding kit was 400 ng for each sample. All mixing steps for DNA samples were performed by gently flicking the microfuge tube instead of pipetting. All libraries were prepared according to the manufacturer’s protocols. The final library was sequenced on R9 SpotON flow cell. The standard 24 h sequencing protocol was initiated using the MinKNOW software (Oxford Nanopore Technologies, Oxford, UK).

Base calling of the raw MinION data was performed with Guppy Basecalling Software version 3.4.4 (Oxford Nanopore Technologies, Oxford, UK), and demultiplexing was made using Guppy barcoding software version 3.4.4 (Oxford Nanopore Technologies, Oxford, UK). Assemblies were obtained using SPAdes version 3.11 [42] (Illumina sequencing) and Unicycler version 0.4.8-beta [43] (hybrid assemblies).

Genome assemblies, including 8 short-read assemblies and 4 hybrid long- and short-read assemblies, were uploaded to NCBI Genbank under the project number PRJNA687166.

### 4.3. Data Processing

Assembled genomes were processed using custom software pipeline including a set of scripts for seamless integration of various available software tools [44]. The main goals of investigations were to determine the antibiotic resistance in silico, to perform isolate typing using various molecular classification schemes, and to reveal the presence of CRISPR/Cas systems in the genomes of the isolated studied. The parameters useful for epidemiological surveillance and the presence of virulence factors were also studied. We used Resfinder 4.0 database for antimicrobial gene identification (https://cge.cbs.dtu.dk/services/ResFinder/, accessed on 20 December 2020).

Analysis of cgMLST loci was performed using MentaList software (https://github.com/WGS-TB/MentaLiST, accessed on 20 December 2020, version 0.2.4), and the tree was build using PHYLOViz online (http://online.phyloviz.net, accessed on 20 December 2020).

CRISPRCasFinder [28] was used to identify the presence of CRISPR/Cas systems and spacers in the genomes studied.

Phylogenetic analyses were conducted using Maximum Likelihood (ML) method in MEGA7.0.26 [45]. The statistical significance of the branches was assessed by bootstrap resampling analysis (1000 replicates).

Prophage sequences prediction and evaluation of the probability of a prophage being active was made using Prophage Hunter (https://pro-hunter.genomics.cn/index.php/Home/Index/index.html, accessed on 20 December 2020) [46].

Direct repeats found in CRISPR arrays were analyzed using CRISPRmapweb tool http://rna.informatik.uni-freiburg.de/CRISPRmap/Input.jsp version v1.3.0-2013, accessed on 20 December 2020, since the human samples were routinely collected, and patients’ data remained anonymous [47,48,49]. 

CRISPR array type was assessed using CRISPRCasdb, where CRISPR4 represents Level 4 CRISPRs (the most reliable ones), while CRISPR levels 1, 2 and 3 may be considered as false CRISPRs [31].

Data analysis and graphing were performed using Prism 9 (GraphPad Software, San Diego, CA, USA).

## Figures and Tables

**Table 1:** The origin and typing of clinical *A. baumannii* isolates studied.

Sample ID	Patient Code	Isolation Date	Clinical Department	Locus	MLST	OCL-Type	KL-Type
CriePir33	A1	03.05.2017	Traumatology	Wound	ST78	OCL1	KL3
CriePir87	A2	04.07.2017	Surgery	Soft tissue abscess	ST2	OCL1	KL33
CriePir158	A3	13.11.2017	Therapy	Sputum	ST45	OCL1	KL49
CriePir168	A4	03.12.2017	ICU anesthesiology	Urine	ST1	OCL1	KL17
CriePir221	A5	21.05.2018	ICU anesthesiology	CVC	ST78	OCL1	KL2
CriePir298	A6	10.09.2019	Rehabilitation of CNS	Urine	ST25	OCL6	KL116
CriePir299	A7	10.04.2019	Neurosurgery	Wound	ST2	OCL1	KL2
CriePir302	A8	21.06.2019	Surgery	Abdominal abscess	ST2	OCL1	KL9
CriePir306	A9	05.08.2019	ICU anesthesiology	BAL	ST15	OCL7	KL9
CriePir307	A10	29.06.2019	ICU anesthesiology	BAL	ST1487 *	OCL2	KL45
CriePir308	A9	22.08.2019	ICU anesthesiology	CVC	ST2	OCL7	KL9
CriePir309	A11	31.08.2019	Rehabilitation of CNS	Urine	ST911	OCL6	KL14

* Novel sequence type found by us is shown in bold; ICU—intensive care unit; CVC—central venous catheter; BAL—bronchoalveolar lavage; the isolates sequenced on MinION are filled with a gray color.

## Data Availability

The data presented in this study are openly available in NCBI Genbank under the project number PRJNA687166. In addition, publicly available datasets were analyzed in this study. This data can also be found in Genbank (https://www.ncbi.nlm.nih.gov/genbank/, accessed on 20 December 2020). Full list of accession numbers is given in Table S3.

## Supplementary Materials

The following are available online at https://www.mdpi.com/2076-0817/10/2/205/s1, Table S1. cgMLST profiles for the isolates studied (pubMLST.org); Table S2. The numbers and features of CRISPR spacers found in *A. baumannii* isolates from multidisciplinary medical center in Moscow, Russia during the period of 2017–2019; Table S3. *A. baumannii* reference isolates; Table S4. Differences in virulence factor frequencies among the *Acinetobacter baumannii* isolates with different CRISPR/Cas systems; Figure S1. Classification of direct repeats of some *A. baumannii* isolates detected in clinical departments of multidisciplinary medical center in Moscow, Russia during the period of 2017–2019 with CRISPRmap web tool; Figure S2. Maximum-likelihood phylogenetic tree of full-length *cas3-cas2* gene sequences of *A. baumannii* detected in clinical departments of multidisciplinary medical center in Moscow, Russia during the period of 2017–2019; Figure S3. Maximum-likelihood phylogenetic tree of full-length *cas6* gene sequences of *A. baumannii* detected in clinical departments of multidisciplinary medical center in Moscow, Russia during the period of 2017–2019; Figure S4. Maximum-likelihood phylogenetic tree of full-length *csy1* gene sequences of *A. baumannii* detected in clinical departments of multidisciplinary medical center in Moscow, Russia during the period of 2017–2019; Figure S5. Maximum-likelihood phylogenetic tree of full-length *csy2* gene sequences of *A. baumannii* detected in clinical departments of multidisciplinary medical center in Moscow, Russia during the period of 2017–2019; Figure S6. Maximum-likelihood phylogenetic tree of full-length *csy3* gene sequences of *A. baumannii* detected in clinical departments of multidisciplinary medical center in Moscow, Russia during the period of 2017–2019; Figure S7. Plasmid number among the *A. baumannii* isolates; Figure S8. Plasmid number among the *A. baumannii* isolates with Type I-FI and TypeI-FII CRISPR/Cas systems; Figure S9. Active prophage sequences among the *A. baumannii* isolates; Figure S10. Low evidence CRISPR arrays among the *A. baumannii* isolates; Figure S11. cgMLST tree for the isolates studied. The numbers of allele differences are indicated.

## References

[1] Munoz-Price L.S., Weinstein R.A. (2008). Acinetobacter infection. N. Engl. J. Med..

[2] Zarrilli R., Giannouli M., Tomasone F., Triassi M., Tsakris A. (2009). Carbapenem resistance in *Acinetobacter baumannii*: The molecular epidemic features of an emerging problem in health care facilities. J. Infect. Dev. Ctries..

[3] World Health Organization (2017). Guidelines for the Prevention and Control of Carbapenem-Resistant Enterobacteriaceae, Acinetobacter Baumannii and Pseudomonas Aeruginosa in Health Care Facilities.

[4] Chebotar I.V., Lazareva A.V., Masalov Y.K., Mikhailovich V.M., Mayanskiy N.A. (2014). Acinetobacter: Microbiological, pathogenetic and resistant properties. Vestn. Ross. Akad. Med. Nauk..

[5] Bartual S.G., Seifert H., Hippler C., Luzon M.A., Wisplinghoff H., Rodriguez-Valera F. (2005). Development of a multilocus sequence typing scheme for characterization of clinical isolates of *Acinetobacter baumannii*. J. Clin. Microbiol..

[6] Diancourt L., Passet V., Nemec A., Dijkshoorn L., Brisse S. (2010). The population structure of *Acinetobacter baumannii*: Expanding multiresistant clones from an ancestral susceptible genetic pool. PLoS ONE.

[7] van Belkum A., Soriaga L.B., LaFave M.C., Akella S., Veyrieras J.B., Barbu E.M., Shortridge D., Blanc B., Hannum G., Zambardi G. (2015). Phylogenetic Distribution of CRISPR-Cas Systems in Antibiotic-Resistant *Pseudomonas aeruginosa*. mBio.

[8] Shariat N., Dudley E.G. (2014). CRISPRs: Molecular signatures used for pathogen subtyping. Appl. Environ. Microbiol..

[9] Fabre L., Zhang J., Guigon G., Le Hello S., Guibert V., Accou-Demartin M., de Romans S., Lim C., Roux C., Passet V. (2012). CRISPR typing and subtyping for improved laboratory surveillance of *Salmonella* infections. PLoS ONE.

[10] Kos V.N., Deraspe M., McLaughlin R.E., Whiteaker J.D., Roy P.H., Alm R.A., Corbeil J., Gardner H. (2015). The resistome of Pseudomonas aeruginosa in relationship to phenotypic susceptibility. Antimicrob. Agents Chemother..

[11] Turton J.F., Wright L., Underwood A., Witney A.A., Chan Y.T., Al-Shahib A., Arnold C., Doumith M., Patel B., Planche T.D. (2015). High-Resolution Analysis by Whole-Genome Sequencing of an International Lineage (Sequence Type 111) of Pseudomonas aeruginosa Associated with Metallo-Carbapenemases in the United Kingdom. J. Clin. Microbiol..

[12] Di Nocera P.P., Rocco F., Giannouli M., Triassi M., Zarrilli R. (2011). Genome organization of epidemic *Acinetobacter baumannii* strains. BMC Microbiol..

[13] Karah N., Samuelsen O., Zarrilli R., Sahl J.W., Wai S.N., Uhlin B.E. (2015). CRISPR-cas subtype I-Fb in *Acinetobacter baumannii*: Evolution and utilization for strain subtyping. PLoS ONE.

[14] Horvath P., Romero D.A., Coute-Monvoisin A.C., Richards M., Deveau H., Moineau S., Boyaval P., Fremaux C., Barrangou R. (2008). Diversity, activity, and evolution of CRISPR loci in *Streptococcus thermophilus*. J. Bacteriol..

[15] Westra E.R., Buckling A., Fineran P.C. (2014). CRISPR-Cas systems: Beyond adaptive immunity. Nat. Rev. Microbiol..

[16] Ershova K., Savin I., Kurdyumova N., Wong D., Danilov G., Shifrin M., Alexandrova I., Sokolova E., Fursova N., Zelman V. (2018). Implementing an infection control and prevention program decreases the incidence of healthcare-associated infections and antibiotic resistance in a Russian neuro-ICU. Antimicrob. Resist. Infect. Control..

[17] Zheng P.X., Chiang-Ni C., Wang S.Y., Tsai P.J., Kuo C.F., Chuang W.J., Lin Y.S., Liu C.C., Wu J.J. (2014). Arrangement and number of clustered regularly interspaced short palindromic repeat spacers are associated with erythromycin susceptibility in emm12, emm75 and emm92 of group A streptococcus. Clin. Microbiol. Infect..

[18] Aydin S., Personne Y., Newire E., Laverick R., Russell O., Roberts A.P., Enne V.I. (2017). Presence of Type I-F CRISPR/Cas systems is associated with antimicrobial susceptibility in *Escherichia coli*. J. Antimicrob. Chemother..

[19] Li H.Y., Kao C.Y., Lin W.H., Zheng P.X., Yan J.J., Wang M.C., Teng C.H., Tseng C.C., Wu J.J. (2018). Characterization of CRISPR-Cas Systems in Clinical *Klebsiella pneumoniae* Isolates Uncovers Its Potential Association with Antibiotic Susceptibility. Front. Microbiol..

[20] Arbatsky N.P., Shneider M.M., Dmitrenok A.S., Popova A.V., Shagin D.A., Shelenkov A.A., Mikhailova Y.V., Edelstein M.V., Knirel Y.A. (2018). Structure and gene cluster of the K125 capsular polysaccharide from *Acinetobacter baumannii* MAR13-1452. Int. J. Biol. Macromol..

[21] Wyres K.L., Cahill S.M., Holt K.E., Hall R.M., Kenyon J.J. (2020). Identification of *Acinetobacter baumannii* loci for capsular polysaccharide (KL) and lipooligosaccharide outer core (OCL) synthesis in genome assemblies using curated reference databases compatible with Kaptive. Microb. Genom..

[22] Shashkov A.S., Cahill S.M., Arbatsky N.P., Westacott A.C., Kasimova A.A., Shneider M.M., Popova A.V., Shagin D.A., Shelenkov A.A., Mikhailova Y.V. (2019). *Acinetobacter baumannii* K116 capsular polysaccharide structure is a hybrid of the K14 and revised K37 structures. Carbohydr. Res..

[23] Shelenkov A., Mikhaylova Y., Yanushevich Y., Samoilov A., Petrova L., Fomina V., Gusarov V., Zamyatin M., Shagin D., Akimkin V. (2020). Molecular Typing, Characterization of Antimicrobial Resistance, Virulence Profiling and Analysis of Whole-Genome Sequence of Clinical *Klebsiella pneumoniae* Isolates. Antibiotics.

[24] Poirel L., Naas T., Nordmann P. (2010). Diversity, epidemiology, and genetics of class D beta-lactamases. Antimicrob. Agents Chemother..

[25] Bonnin R.A., Potron A., Poirel L., Lecuyer H., Neri R., Nordmann P. (2011). PER-7, an extended-spectrum beta-lactamase with increased activity toward broad-spectrum cephalosporins in *Acinetobacter baumannii*. Antimicrob. Agents Chemother..

[26] Wang H., Guo P., Sun H., Wang H., Yang Q., Chen M., Xu Y., Zhu Y. (2007). Molecular epidemiology of clinical isolates of carbapenem-resistant Acinetobacter spp. from Chinese hospitals. Antimicrob. Agents Chemother..

[27] Jia H., Sun Q., Ruan Z., Xie X. (2019). Characterization of a small plasmid carrying the carbapenem resistance gene bla OXA-72 from community-acquired *Acinetobacter baumannii* sequence type 880 in China. Infect. Drug Resist..

[28] Couvin D., Bernheim A., Toffano-Nioche C., Touchon M., Michalik J., Neron B., Rocha E.P.C., Vergnaud G., Gautheret D., Pourcel C. (2018). CRISPRCasFinder, an update of CRISRFinder, includes a portable version, enhanced performance and integrates search for Cas proteins. Nucleic Acids Res..

[29] Makarova K.S., Wolf Y.I., Iranzo J., Shmakov S.A., Alkhnbashi O.S., Brouns S.J.J., Charpentier E., Cheng D., Haft D.H., Horvath P. (2020). Evolutionary classification of CRISPR-Cas systems: A burst of class 2 and derived variants. Nat. Rev. Microbiol..

[30] Koonin E.V., Makarova K.S., Zhang F. (2017). Diversity, classification and evolution of CRISPR-Cas systems. Curr. Opin. Microbiol..

[31] Pourcel C., Touchon M., Villeriot N., Vernadet J.P., Couvin D., Toffano-Nioche C., Vergnaud G. (2020). CRISPRCasdb a successor of CRISPRdb containing CRISPR arrays and cas genes from complete genome sequences, and tools to download and query lists of repeats and spacers. Nucleic Acids Res..

[32] Shelenkov A., Korotkov A., Korotkov E. (2008). MMsat—A database of potential micro- and minisatellites. Gene.

[33] Yeh H.Y., Awad A. (2020). Genotyping of *Campylobacter jejuni* Isolates from Poultry by Clustered Regularly Interspaced Short Palindromic Repeats (CRISPR). Curr. Microbiol..

[34] Shelenkov A., Korotkov E. (2009). Search of regular sequences in promoters from eukaryotic genomes. Comput. Biol. Chem..

[35] Tuminauskaite D., Norkunaite D., Fiodorovaite M., Tumas S., Songailiene I., Tamulaitiene G., Sinkunas T. (2020). DNA interference is controlled by R-loop length in a type I-F1 CRISPR-Cas system. BMC Biol..

[36] Fagerlund R.D., Wilkinson M.E., Klykov O., Barendregt A., Pearce F.G., Kieper S.N., Maxwell H.W.R., Capolupo A., Heck A.J.R., Krause K.L. (2017). Spacer capture and integration by a type I-F Cas1-Cas2-3 CRISPR adaptation complex. Proc. Natl. Acad. Sci. USA.

[37] Rollins M.F., Chowdhury S., Carter J., Golden S.M., Wilkinson R.A., Bondy-Denomy J., Lander G.C., Wiedenheft B. (2017). Cas1 and the Csy complex are opposing regulators of Cas2/3 nuclease activity. Proc. Natl. Acad. Sci. USA.

[38] Zheng Y., Li J., Wang B., Han J., Hao Y., Wang S., Ma X., Yang S., Ma L., Yi L. (2020). Endogenous Type I CRISPR-Cas: From Foreign DNA Defense to Prokaryotic Engineering. Front. Bioeng. Biotechnol..

[39] Pausch P., Muller-Esparza H., Gleditzsch D., Altegoer F., Randau L., Bange G. (2017). Structural Variation of Type I-F CRISPR RNA Guided DNA Surveillance. Mol. Cell.

[40] Palmer K.L., Gilmore M.S. (2010). Multidrug-resistant enterococci lack CRISPR-cas. mBio.

[41] Mangas E.L., Rubio A., Alvarez-Marin R., Labrador-Herrera G., Pachon J., Pachon-Ibanez M.E., Divina F., Perez-Pulido A.J. (2019). Pangenome of *Acinetobacter baumannii* uncovers two groups of genomes, one of them with genes involved in CRISPR/Cas defence systems associated with the absence of plasmids and exclusive genes for biofilm formation. Microb. Genom..

[42] Bankevich A., Nurk S., Antipov D., Gurevich A.A., Dvorkin M., Kulikov A.S., Lesin V.M., Nikolenko S.I., Pham S., Prjibelski A.D. (2012). SPAdes: A new genome assembly algorithm and its applications to single-cell sequencing. J. Comput. Biol..

[43] Wick R.R., Judd L.M., Gorrie C.L., Holt K.E. (2017). Unicycler: Resolving bacterial genome assemblies from short and long sequencing reads. PLoS Comput. Biol..

[44] Shelenkov A., Petrova L., Fomina V., Zamyatin M., Mikhaylova Y., Akimkin V. (2020). Multidrug-Resistant *Proteus mirabilis* Strain with Cointegrate Plasmid. Microorganisms.

[45] Kumar S., Stecher G., Tamura K. (2016). MEGA7: Molecular Evolutionary Genetics Analysis Version 7.0 for Bigger Datasets. Mol. Biol. Evol..

[46] Song W., Sun H.X., Zhang C., Cheng L., Peng Y., Deng Z., Wang D., Wang Y., Hu M., Liu W. (2019). Prophage Hunter: An integrative hunting tool for active prophages. Nucleic Acids Res..

[47] Alkhnbashi O.S., Costa F., Shah S.A., Garrett R.A., Saunders S.J., Backofen R. (2014). CRISPRstrand: Predicting repeat orientations to determine the crRNA-encoding strand at CRISPR loci. Bioinformatics.

[48] Lange S.J., Alkhnbashi O.S., Rose D., Will S., Backofen R. (2013). CRISPRmap: An automated classification of repeat conservation in prokaryotic adaptive immune systems. Nucleic Acids Res..

[49] Raden M., Ali S.M., Alkhnbashi O.S., Busch A., Costa F., Davis J.A., Eggenhofer F., Gelhausen R., Georg J., Heyne S. (2018). Freiburg RNA tools: A central online resource for RNA-focused research and teaching. Nucleic Acids Res..

