# Supplementary material for: Genomic and Phenotypic Analysis of Multidrug-Resistant Acinetobacter baumannii Clinical Isolates Carrying Different Types of CRISPR/Cas Systems
Source: Pathogens. 2021 Feb 13;10(2):205. doi: 10.3390/pathogens10020205 (PMC7918744; doi:10.3390/pathogens10020205)
Supplement: Supplementary file 1 [file pathogens-10-00205-s001.zip › figures_suppl_R1.pdf]

Supplementary data

Genomic and phenotypic analysis of multidrug-resistant *Acinetobacter baumannii* clinical isolates carrying different types of CRISPR/Cas systems

Marina Tyumentseva[1 ϒ], Yulia Mikhaylova[1 ϒ], Anna Prelovskaya[1], Aleksandr Tyumentsev [1], Lyudmila Petrova[2], Valeria Fomina[2], Mikhail Zamyatin[2], Andrey Shelenkov[1,*] and Vasiliy Akimkin[1]

[1] Central Research Institute of Epidemiology, Novogireevskaya str., 3a, 111123 Moscow, Russia;

[2] National Medical and Surgical Center named after N.I. Pirogov, Nizhnyaya Pervomayskaya str., 70, 105203 Moscow, Russia

ϒ – authors contributed equally

* - corresponding author

<table>
<tr><td colspan="2">P-168 and P-298</td></tr>
<tr><td>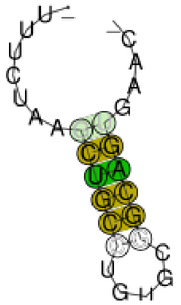
motif 6</td><td>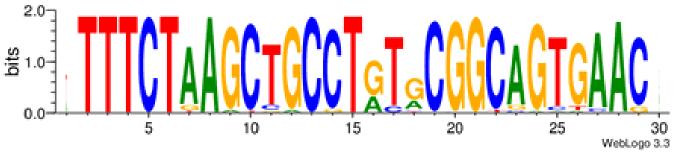

family 8</td></tr>
<tr><td colspan="2">P-307</td></tr>
<tr><td>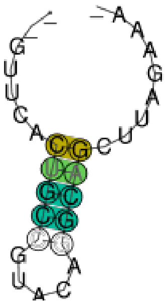
motif 4</td><td>unclassified</td></tr>
</table>

Figure S1. Classification of direct repeats of some *A. baumannii* isolates detected in clinical departments of multidisciplinary medical center in Moscow, Russia during the period of 2017-2019 with CRISPRmap web tool (http://rna.informatik.uni-freiburg.de/CRISPRmap/Input.jsp).

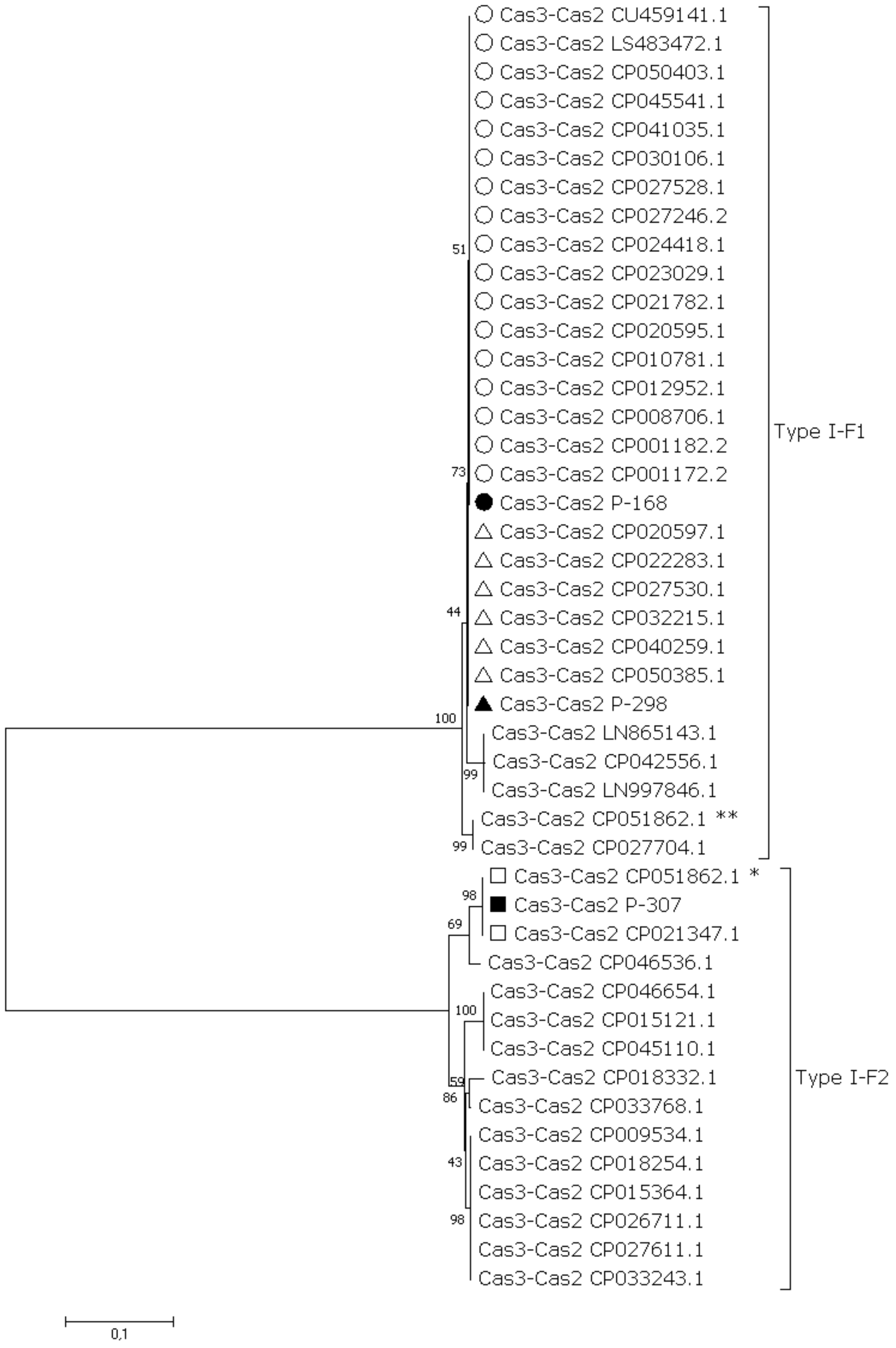

Figure S2. Maximum-likelihood phylogenetic tree of full-length *cas3-cas2* gene sequences of *A. baumannii* detected in clinical departments of multidisciplinary medical center in Moscow, Russia during the period of 2017-2019. Bootstrap test (1000 replicates) was used, and bootstrap values are shown at the branch nodes. The sequences identified in this study are marked with black circle, square and triangle. Reference sequences are marked with transparent circles, squares and triangles. Sequences identified in this study are indicated by strain name, reference sequences – by GenBank accession number. Asterisks indicate two different cassettes of *cas* genes found in one of the reference isolates.

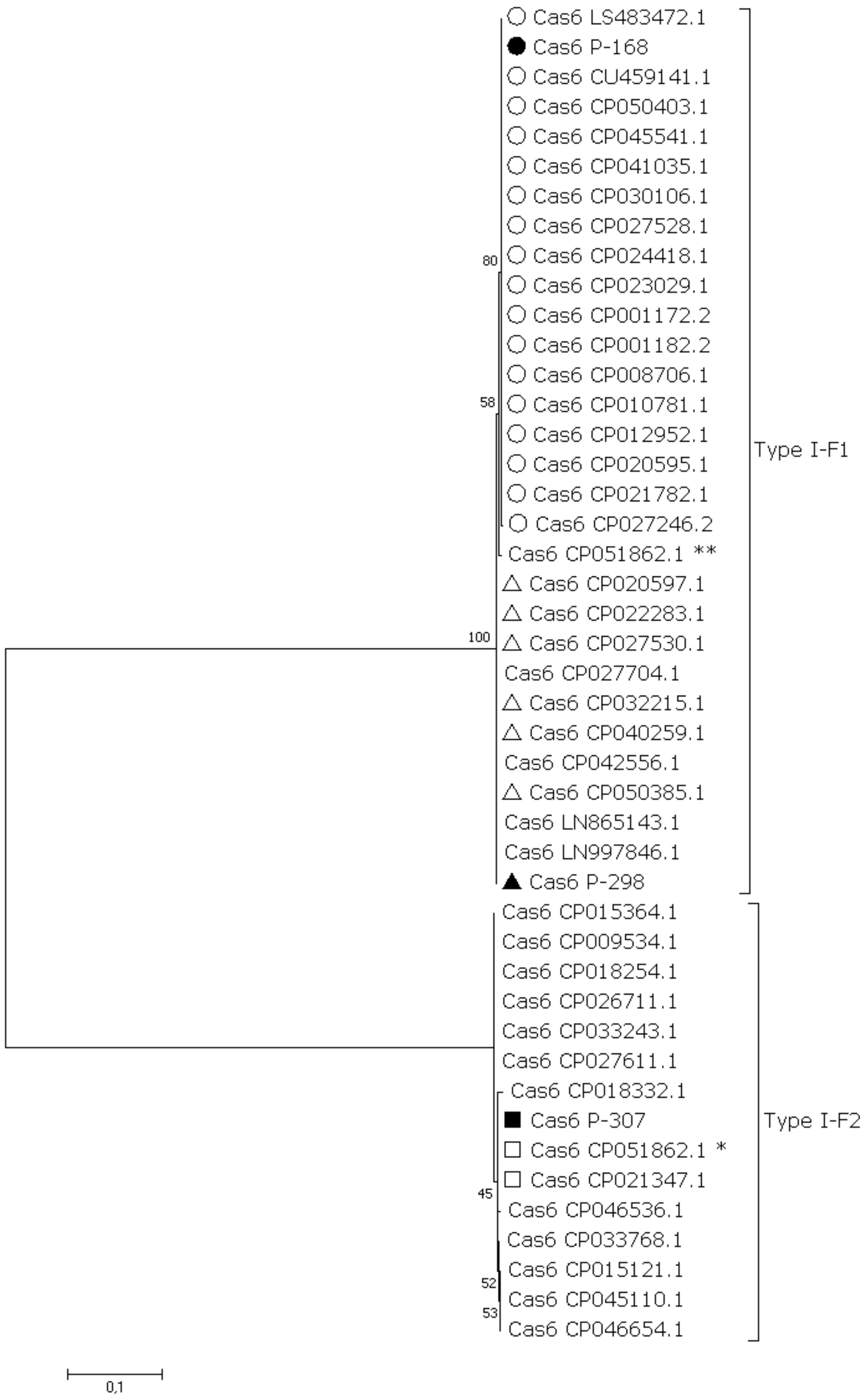

Figure S3. Maximum-likelihood phylogenetic tree of full-length *cas6* gene sequences of *A. baumannii* detected in clinical departments of multidisciplinary medical center in Moscow, Russia during the period of 2017-2019. Bootstrap test (1000 replicates) was used, and bootstrap values are shown at the branch nodes. The sequences identified in this study are marked with black circle, square and triangle. Reference sequences are marked with transparent circles, squares and triangles. Sequences identified in this study are indicated by strain name, reference sequences – by GenBank accession number. Asterisks indicate two different cassettes of *cas* genes, found in one of the reference isolates.

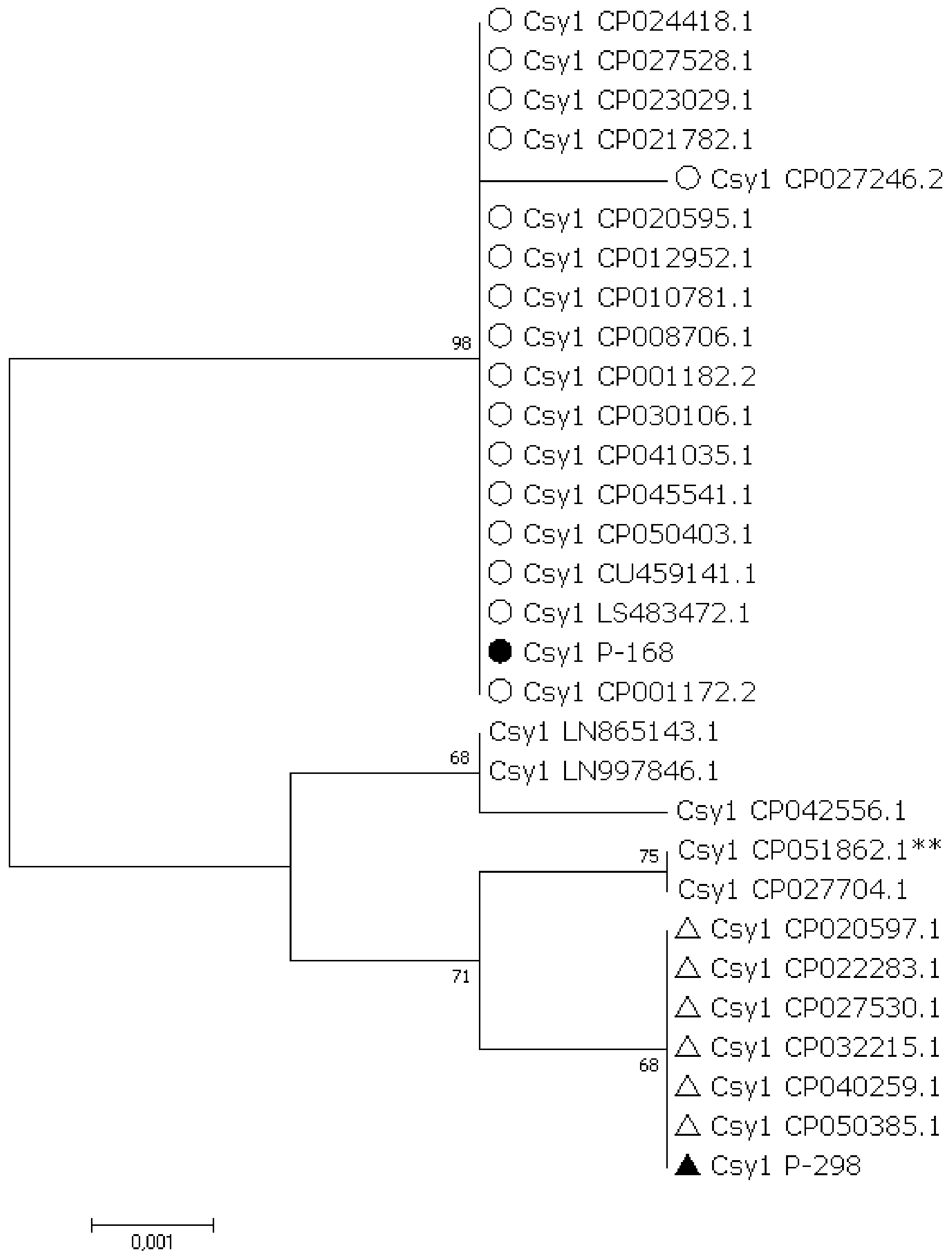

Figure S4. Maximum-likelihood phylogenetic tree of full-length *csy1* gene sequences of *A. baumannii* detected in clinical departments of multidisciplinary medical center in Moscow, Russia during the period of 2017-2019. Bootstrap test (1000 replicates) was used, and bootstrap values are shown at the branch nodes. The sequences identified in this study are marked with black circle, square and triangle. Reference sequences are marked with transparent circles, squares and triangles. Sequences identified in this study are indicated by strain name, reference sequences – by GenBank accession number. Asterisks indicate two different cassettes of *cas* genes, found in one of the reference isolates.

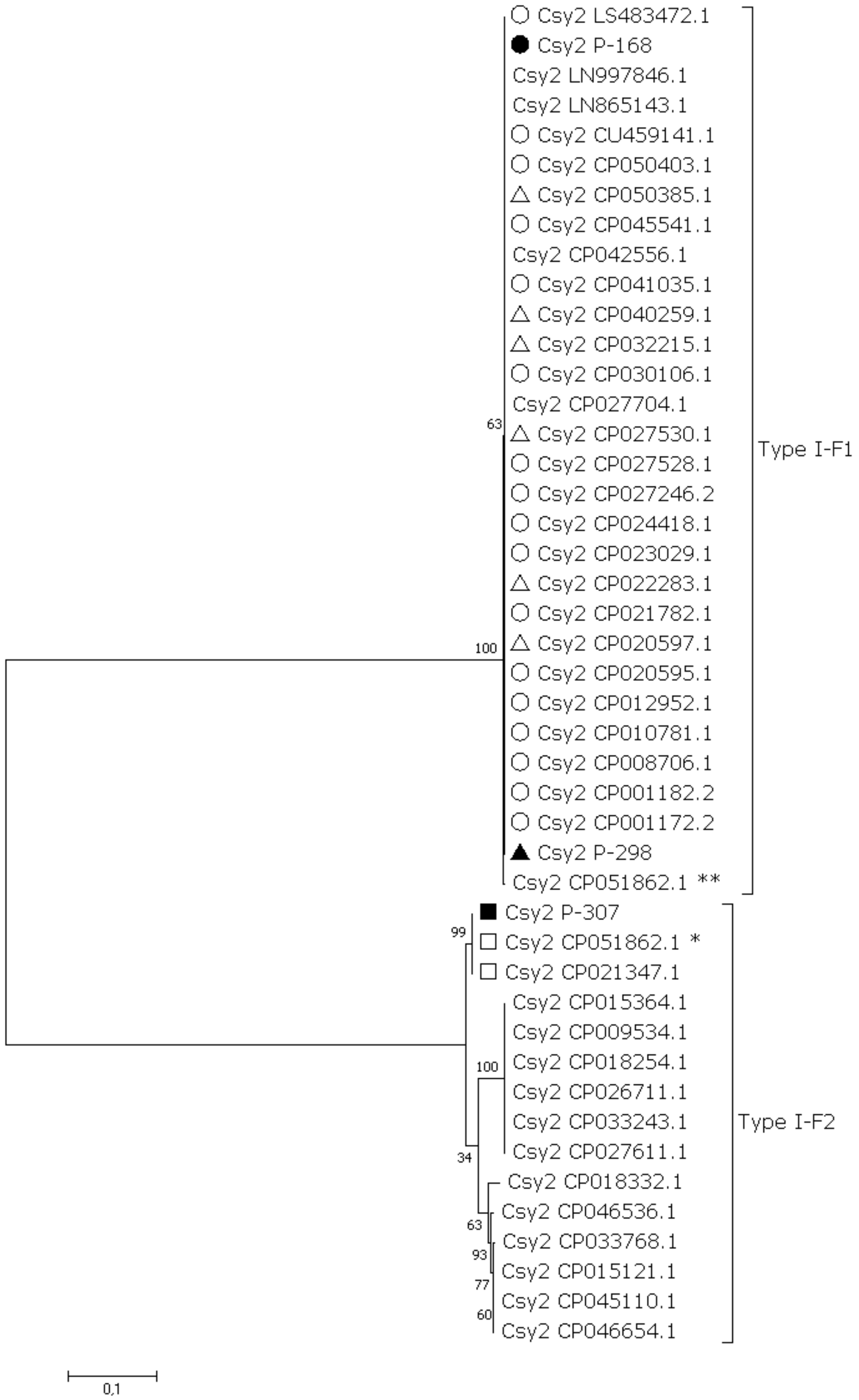

Figure S5. Maximum-likelihood phylogenetic tree of full-length *csy2* gene sequences of *A. baumannii* detected in clinical departments of multidisciplinary medical center in Moscow, Russia during the period of 2017-2019. Bootstrap test (1000 replicates) was used, and bootstrap values are shown at the branch nodes. The sequences identified in this study are marked with black circle, square and triangle. Reference sequences are marked with transparent circles, squares and triangles. Sequences identified in this study are indicated by strain name, reference sequences – by GenBank accession number. Asterisks indicate two different cassettes of *cas* genes, found in one of the reference isolates.

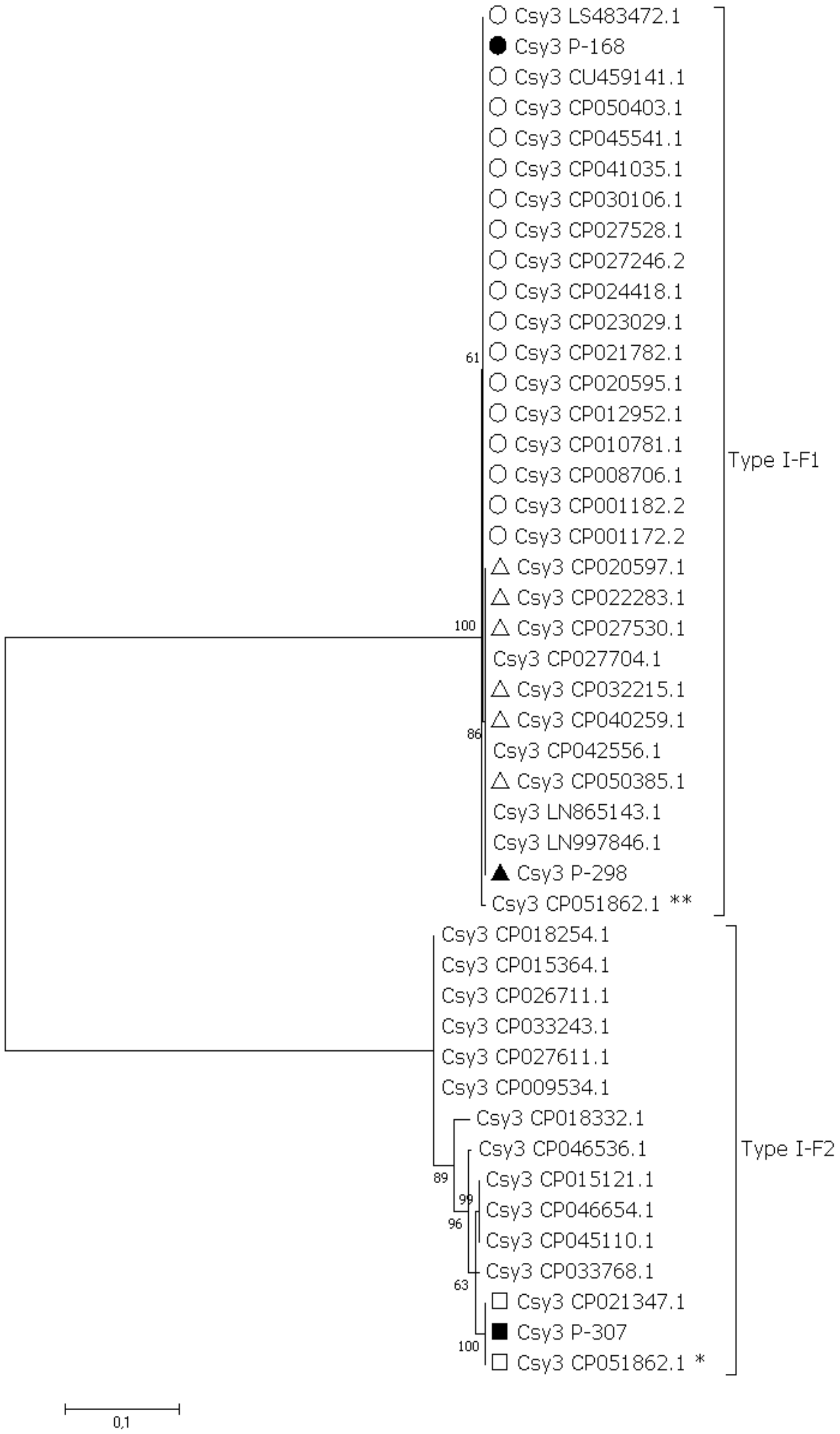

Figure S6. Maximum-likelihood phylogenetic tree of full-length *csy3* gene sequences of *A. baumannii* detected in clinical departments of multidisciplinary medical center in Moscow, Russia during the period of 2017-2019. Bootstrap test (1000 replicates) was used, and bootstrap values are shown at the branch nodes. The sequences identified in this study are marked with black circle, square and triangle. Reference sequences are marked with transparent circles, squares and triangles. Sequences identified in this study are indicated by strain name, reference sequences – by GenBank accession number. Asterisks indicate two different cassettes of *cas* genes found in one of the reference isolates.

Figure S7. **Plasmids among the *Acinetobacter baumannii* isolates**

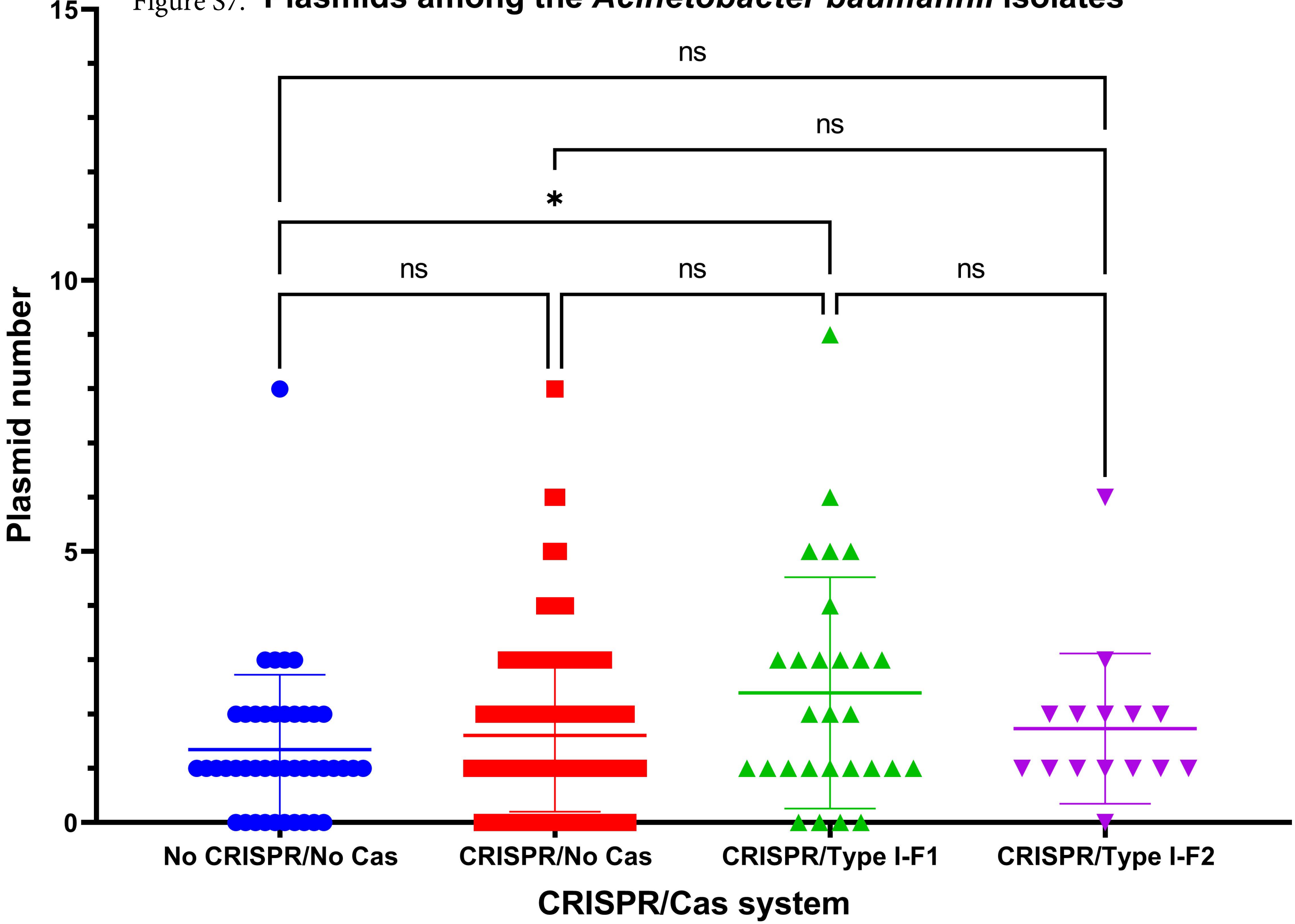

Plasmids among the *Acinetobacter baumannii* isolates

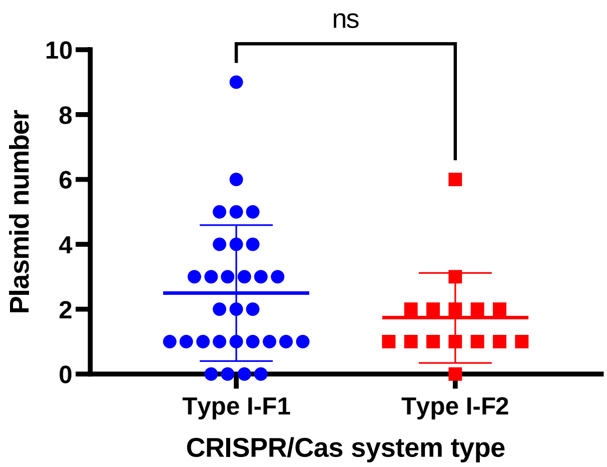

P value	0.2077
P value summary	ns
Significantly different (P < 0.05)?	No

Figure S8. Plasmid number among the A. baumannii isolates with Type I-FI and Type I-FII CRISPR/Cas systems

Active prophages among the *Acinetobacter baumannii* isolates

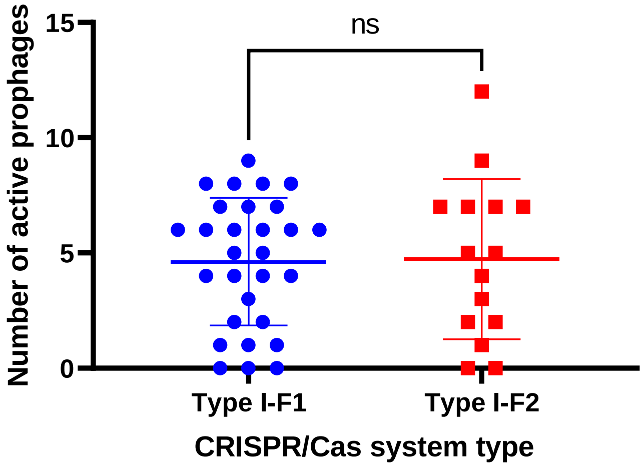

P value	0.9073
P value summary	ns
Significantly different (P < 0.05)?	No

Figure S9. Active prophage sequences among the *A. baumannii* isolates.

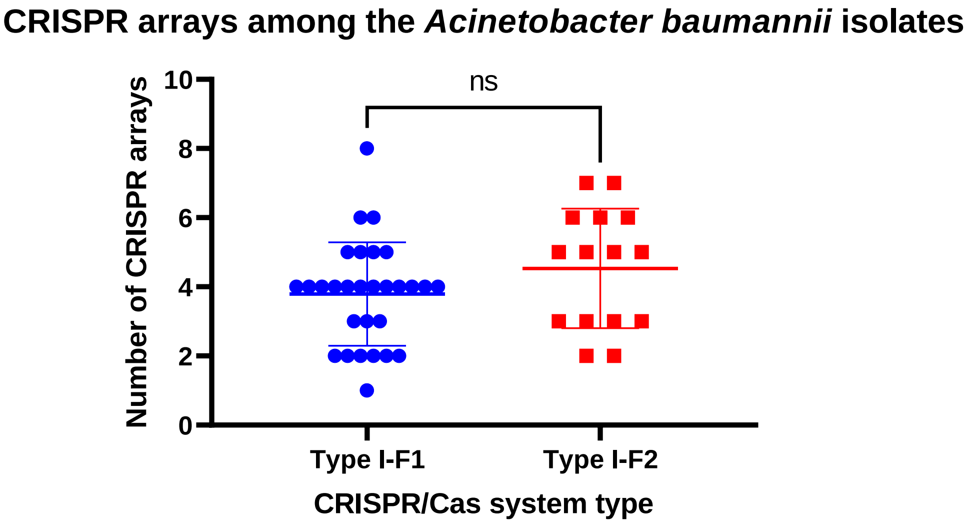

P value	0.1475
P value summary	ns
Significantly different (P < 0.05)?	No

Figure S10. Low evidence CRISPR arrays among the *A. baumannii* isolates.

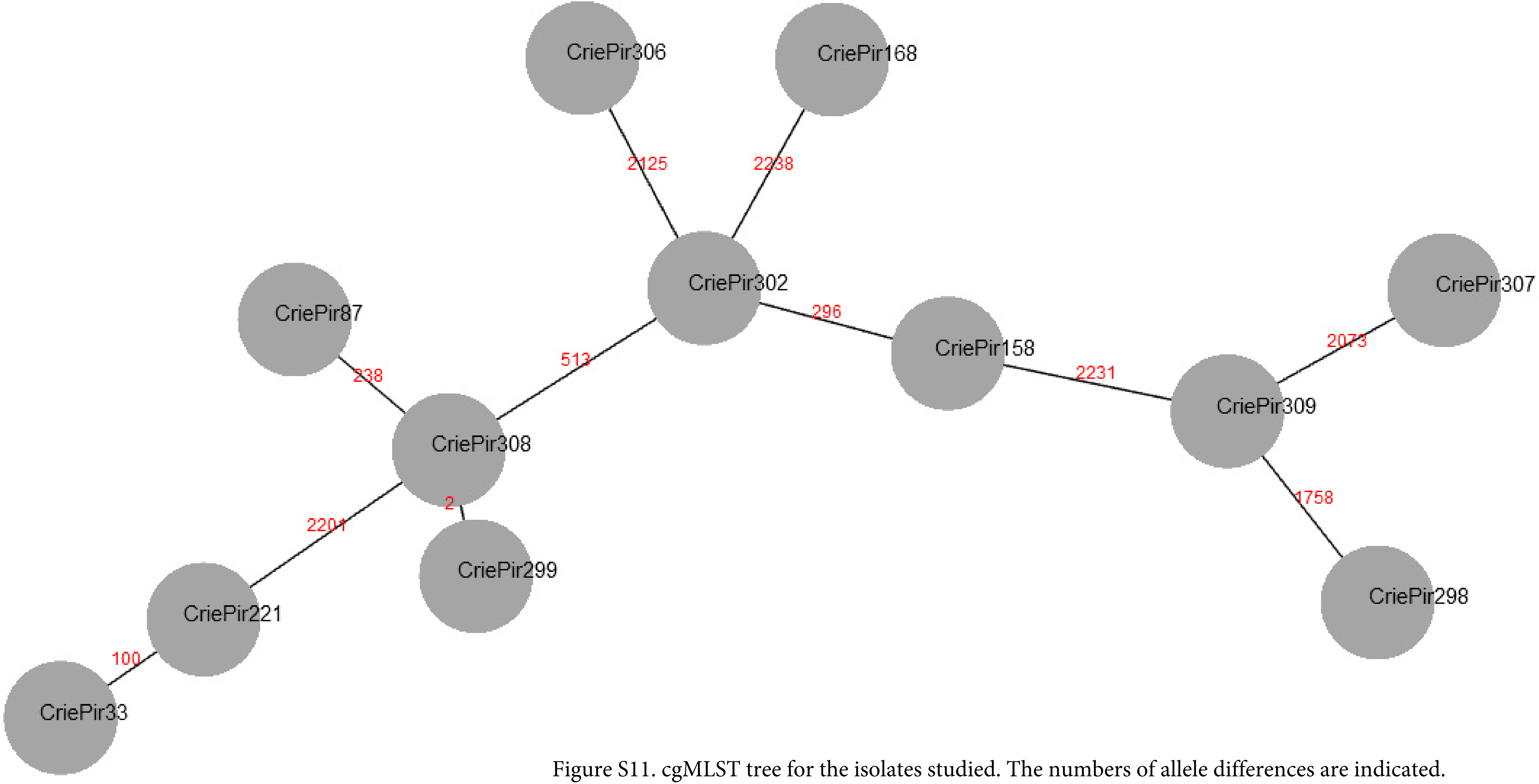

Figure S11. cgMLST tree for the isolates studied. The numbers of allele differences are indicated.
